# Hepatocellular Carcinoma Developing in a Patient 29 Years After Achieving Sustained Virologic Response for Hepatitis C With Interferon Therapy: A Case Report

**DOI:** 10.7759/cureus.74330

**Published:** 2024-11-23

**Authors:** Yosuke Maezawa, Yukiko Kodama, Hiroyuki Ariga, Junya Kashimura, Toshiyuki Irie

**Affiliations:** 1 Department of Internal Medicine, Mito Kyodo General Hospital, Mito, JPN; 2 Department of Gastroenterology, Mito Kyodo General Hospital, Mito, JPN; 3 Department of Radiology, Mito Kyodo General Hospital, Mito, JPN

**Keywords:** direct-acting antiviral drugs, hepatitis c virus (hcv), hepatocellular carcinoma (hcc), interferon therapy, sustained virologic response (svr)

## Abstract

We report a case of an 87-year-old female with a ruptured hepatocellular carcinoma (HCC). She presented with sudden epigastric and right upper abdominal pain. The physical examination revealed mild tenderness in the right upper abdomen, a positive Murphy's sign, and no jaundice. Laboratory tests showed mild anemia and elevated PIVKA-II (prothrombin induced by vitamin K absence II) levels. Abdominal ultrasound and CT revealed a large hypervascular mass in the liver, along with ascites and portal vein thrombosis.

The patient had received interferon therapy for hepatitis C virus (HCV) 29 years before her presentation and blood tests and imaging examinations had confirmed sustained undetectability for HCV ribonucleic acid (RNA) and the absence of HCC for five years following treatment. HCV RNA remained undetectable at the time of her admission, and it was presumed that it had been negative for 29 years post-treatment, with no evidence of re-exposure. The patient had not attended any follow-up appointments. While there have been no reported cases of a patient developing HCC 29 years after achieving sustained virologic response (SVR), our case suggests that the absence of HCC risk is not guaranteed, even after a prolonged period post-SVR. Therefore, periodic imaging tests such as abdominal ultrasound or CT may be beneficial in detecting potential HCC, even long after achieving SVR.

## Introduction

Hepatocellular carcinoma (HCC) accounts for 85-90% of all primary liver cancers. Approximately half a million people worldwide are newly diagnosed with HCC annually, and a similar number die from this disease. Chronic hepatitis B virus (HBV) and hepatitis C virus (HCV) infections are responsible for the majority of HCC cases [1,2]. Historically, interferon-based therapy had been the mainstay of treatment for HCV. However, around 2011, treatment strategies shifted towards direct-acting antiviral drugs (DAAs) [3], enabling sustained virologic response (SVR) in many patients. SVR is defined as the absence of detectable HCV RNA 12 weeks or more after completing antiviral treatment. However, HCC can still occur after achieving SVR, but there is currently no definitive, evidence-based method to assess this risk [4]. We present a case of a patient who developed HCC 29 years after successful interferon therapy for HCV. To the best of our knowledge, there have been no reported cases of HCC developing after such a prolonged period post-SVR, and hence this report highlights a unique clinical scenario.

## Case presentation

The patient was an 87-year-old female who had visited an urgent care unit in the evening due to sudden pain in the epigastric region and right upper abdomen and had then been referred to the emergency department of our hospital the same day for further investigation and treatment. She had received interferon therapy for hepatitis C at another hospital 29 years earlier, and her blood HCV RNA levels had remained negative following therapy. The patient visited our hospital independently. Her vital signs were as follows - blood pressure: 150/70 mmHg, pulse rate: 63/min, respiratory rate: 15/min, SpO_2_: 96% (on room air), and body temperature: 35.9 °C. Physical examination revealed clear consciousness, pallor, and no jaundice in the eyelids or conjunctiva. She had mild tenderness in the pericardial area of the right upper abdomen, with a positive Murphy's sign. There was no pain on percussion over the hepatic area and no signs of peritoneal irritation. She was not taking any medication and had no history of smoking or alcohol consumption.

Blood tests revealed anemia, with Hb of 9.2 g/dL, but no elevated inflammatory response, hepatobiliary or pancreatic enzyme abnormalities, abnormal coagulation, or glucose intolerance. As for hepatitis viruses, HCV antibodies were positive but HCV RNA was not detected, and HBV was negative. Tumor markers, including α-fetoprotein (AFP), were within the normal range at 1.2 ng/mL, but PIVKA-II (prothrombin induced by vitamin K absence II) was elevated at 11,200 mAU/mL. The fibrosis markers, type IV collagen, and hyaluronic acid were only mildly elevated at 166 ng/mL and 109 ng/mL, respectively. Both anti-nuclear antibodies and anti-mitochondrial antibodies were negative (Table 1).

**Table 1:** Laboratory test results on admission There was mild anemia and slightly elevated levels of AST and LDH, while HCV RNA was not detected

Variables	Result	Reference range
White blood cells, /µL	5,900	3,300-8,600
Hemoglobin, g/dL	9.2	11.6-14.8
Platelets, /µL	16.6 ×10^4^	15.8-34.8 ×10^4^
Prothrombin time, %	100.1	70-130
Activated partial thromboplastin time (APTT), sec	27	24.3-36.0
Total protein, g/dL	6.4	6.6-8.1
Albumin, g/dL	3.7	4.1-5.1
Aspartate aminotransferase (AST), IU/L	35	13-30
Alanine aminotransferase (ALT), IU/L	15	7-23
Lactate dehydrogenase (LDH), IU/L	307	124-222
Alkaline phosphatase (ALP), IU/L	172	106-322
γ-glutamyl transpeptidase (GGT), IU/L	27	9-32
Amylase, IU/L	79	44-132
Total bilirubin, mg/dL	0.6	0.4-1.5
Blood urea nitrogen, mg/dL	24	8-20
Creatinine, mg/dL	1.18	0.46-0.79
Sodium, mEq/L	143	138-145
Chloride, mEq/L	105	101-108
Pottasium, mEq/L	4.1	3.6-4.8
Calcium, mg/dL	8.8	8.8-10.1
C-reactive protein (CRP), mg/dL	0.02	0.00-0.14
Blood glucose level, mg/dL	127	70-109
HbA1c, %	5.1	4.6-6.2
HBs antigen	Negative	Negative
HBs antibody	Negative	Negative
HBc antibody	Negative	Negative
HBV DNA	Not detected	Not detected
HCV antibody	Positive	Negative
HCV RNA	Not detected	Not detected
α-fetoprotein (AFP), ng/mL	1.2	<10.0
Prothrombin induced by vitamin K absence II (PIVKA-II), mAU/mL	11,200	<40.0
Carcinoembryonic antigen (CEA), ng/mL	2.8	<5.0
Carbohydrate antigen 19-9 (CA19-9), U/mL	14.4	<37.0
Type IV collagen, ng/mL	166	<150
Hyaluronic acid, ng/mL	109	<50

**Figure 1:**
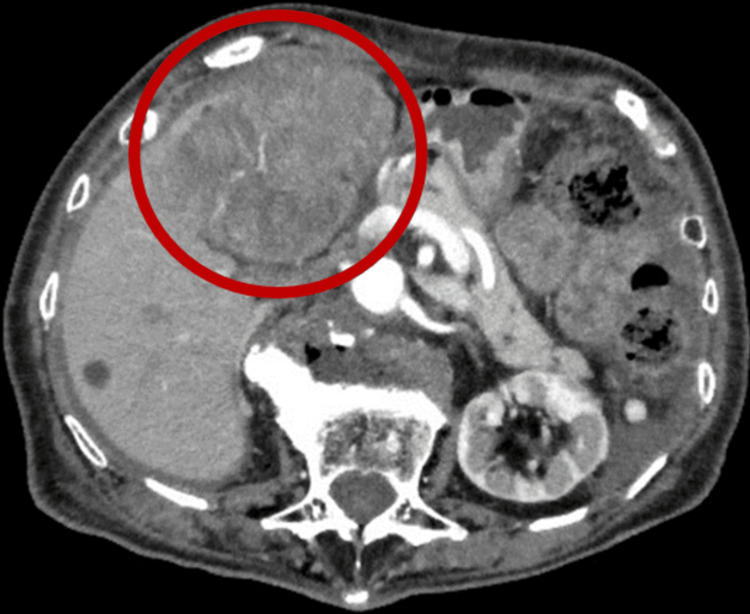
Dynamic CT of the abdomen revealing an 82 × 63 mm hypervascular mosaic mass in the S4 area of the liver (red circle) CT: computed tomography

**Figure 2:**
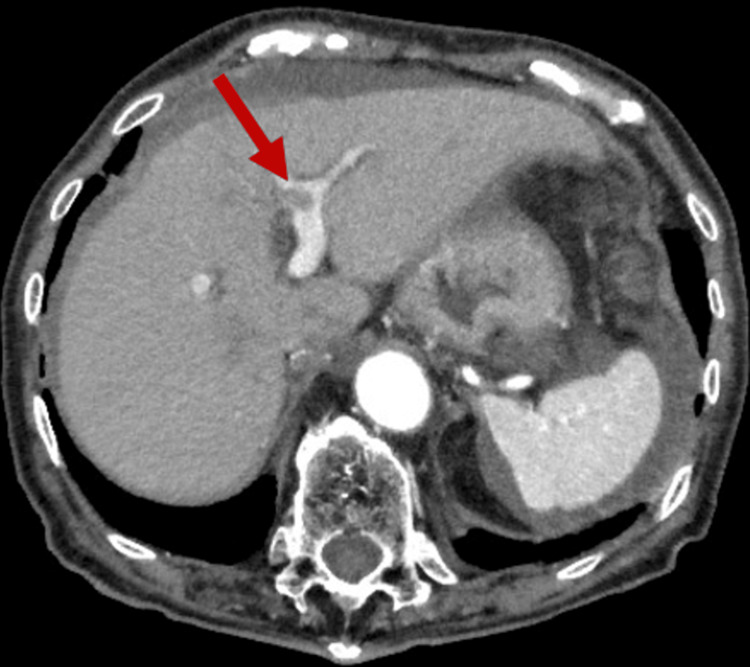
CT revealing ascites around the liver and a tumor thrombus in the portal vein (red arrow) CT: computed tomography

Abdominal ultrasound revealed an intrahepatic mass with a diameter of approximately 80 mm and ascitic effusion, mainly around the liver. Dynamic CT of the abdomen revealed an 82 × 63 mm hypervascular mosaic mass in the S4 area of the liver (Figure 1). There was no contrast extravasation and a small area of ascites with a high-density indicating hematoma was observed around the periportal area of the liver. A tumor thrombosis was observed in the portal vein (Figure 2). We did not perform pathological examinations; however, we concluded that the CT findings were consistent with a diagnosis of HCC.

**Figure 3:**
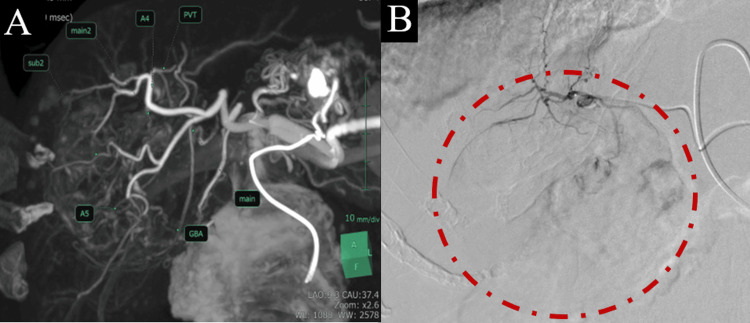
TACE performed on the day of admission for ruptured HCC A: Confirmation of the arterial anatomy with 3D CT angiography. B: Embolization of the right hepatic artery responsible for the ruptured HCC (red circle) CT: computed tomography; HCC: hepatocellular carcinoma; TACE: transcatheter arterial chemoembolization

The patient was diagnosed with ruptured HCC and was admitted for emergency hospitalization. Transcatheter arterial chemoembolization (TACE) was performed on the day of admission (Figure 3). A month after TACE, CT revealed a marked reduction in tumor size. However, due to the patient’s declining health and independence during the hospitalization, after obtaining informed consent from her family, we decided not to pursue additional treatment. She was transferred to a rehabilitation facility 31 days after the admission.

## Discussion

We discussed a case of ruptured HCC where interferon therapy had been administered for hepatitis C, achieving HCV RNA negativity. During the initial treatment period, blood tests and imaging had been performed over five years to confirm the absence of chronic hepatitis C or HCC development, leading to the conclusion of follow-up. However, periodic imaging examinations had not been performed afterward. HCC commonly develops against a background of liver cirrhosis or fibrosis. Although a liver biopsy was not performed, CT imaging showed no signs of liver cirrhosis or splenomegaly, and blood tests revealed no findings suggestive of cirrhosis. To screen for autoimmune hepatitis or primary biliary cholangitis, we measured anti-nuclear and anti-mitochondrial antibodies, both of which were negative. In cases of HCC without clear causes, chronic liver disease, such as hepatitis B or C virus infection, may have been present in the past [5].

Achieving sustained HCV RNA negativity in chronic hepatitis and cirrhosis caused by HCV reduces the risk of HCC [6-9]. Several studies have shown that old age, male sex, progression of fibrosis, diabetes mellitus, and elevated AFP levels are significant risk factors [10-13]. Comparing interferon-treated patients with those treated with DAAs - now the primary treatment for hepatitis C - showed no significant difference in adjusted HCC risk after accounting for age and observation period [14]. In our case, although the patient was older, other risk factors, such as male sex, fibrosis progression, diabetes, and elevated AFP levels, were not present.

Following treatment, the patient did not undergo regular medical check-ups. HCC developed in her despite HCV RNA negativity and the absence of cirrhosis. There were no identified risk factors for developing HCC other than hepatitis C, such as HBV infection, a history of alcohol consumption, smoking, or diabetes. Although achieving SVR with antiviral therapy suppresses the development of HCC, the risk of HCC does not completely disappear. After achieving SVR, the reported five- and 10-year incidence rates of HCC range from 2.3% to 8.8% and 3.1% to 11.1%, respectively [15]. Although guidelines do not explicitly specify a follow-up period after confirming sustained HCV RNA negativity, the risk of HCC remains, suggesting that periodic imaging surveillance may be beneficial.

Currently, there are no prospective studies that directly investigate the effective methods and utility of HCC screening after achieving SVR, and its cost-effectiveness remains unclear. However, retrospective studies in Japan have suggested a potential improvement in prognosis with regular imaging examinations. In cases where abdominal ultrasound examinations were conducted at least every six months, the five-year survival rate was 93%, compared to 60% in cases without such screenings [16]. While further research is needed to determine its direct impact on prognosis, periodic follow-up appears to have significant potential for improving outcomes.

## Conclusions

Over the past decade, the primary treatment for hepatitis C has shifted from interferon-based therapies to DAAs. However, there is no significant difference in the risk of developing HCC between interferon and DAA treatments. While achieving SVR after completing treatment significantly reduces the incidence of HCC compared to untreated cases, it does not fully eliminate the risk. Even if treatment is conducted according to the guidelines, there is a strong possibility that follow-up will be concluded after five or 10 years of post-treatment observation. However, as in our case, there is a risk that HCC could occur after an even longer period has elapsed. Therefore, regular screening, such as abdominal ultrasound or CT, needs to be performed in these patients to confirm the absence of HCC.
